# Perceptions of Chinese hospital leaders on joint commission international accreditation: a qualitative study

**DOI:** 10.3389/fpubh.2023.1258600

**Published:** 2023-10-30

**Authors:** HongFan Zhang, Siou-Tang Huang, Mark J. Bittle, Lilly Engineer, Herng-Chia Chiu

**Affiliations:** ^1^Department of Health Policy and Management, Johns Hopkins Bloomberg School of Public Health, Johns Hopkins University, Baltimore, MD, United States; ^2^Institute for Hospital Management, Tsinghua University, Shenzhen, China; ^3^Department of Anesthesiology and Critical Care Medicine, School of Medicine, Johns Hopkins University, Baltimore, MD, United States; ^4^Department of Healthcare Administration and Medical Informatics, Kaohsiung Medical University, Kaohsiung, Taiwan

**Keywords:** joint commission international, accreditation, leadership, Chinese hospital, qualitative study

## Abstract

**Background:**

Joint Commission International (JCI) accreditation plays a significant role in improving the quality of care and patient safety worldwide. Hospital leadership is critical in making international accreditation happen with successful implementation. Little is known about how Chinese hospital leaders experienced and perceived the impact of JCI accreditation. This paper is the first study to explore the perceptions of hospital leaders toward JCI accreditation in China.

**Methods:**

Qualitative semi-structured interviews were used to explore the perceptions of the chief operating officers, the chief medical officers, and the chief quality officers in five JCI-accredited hospitals in China. Thematic analysis was used to analyze the interview transcripts and identify the main themes.

**Results:**

Fifteen hospital leaders participated in the interviews. Three themes emerged from the analysis, namely the motivations, challenges, and benefits related to pursuing and implementing JCI accreditation. The qualitative study found that eight factors influenced hospital leadership to pursue JCI accreditation, five challenges were identified with implementing JCI standards, and eight benefits emerged from the leadership perspective.

**Conclusion:**

Pursuing JCI accreditation is a discretionary decision by the hospital leadership. Participants were motivated by prevalent perceptions that JCI requirements would be used as a management tool to improve the quality of care and patient safety in their hospitals. These same organizational leaders identified challenges associated with implementing and sustaining JCI accreditation. The significant challenges were a clear understanding of the foreign accreditation standards, making staff actively participate in JCI processes, and changing staff behaviors accordingly. The top 5 perceived benefits to JCI accreditation from the leaders’ perspective were improved leadership and hospital safety, improvements in the care processes, and the quality of care and the learning culture improved. Other perceived benefits include enhanced reputation, better cost containment, and a sense of pride in the staff in JCI-accredited hospitals.

## Introduction

A healthcare accreditation program may serve as an external means to assess or improve the quality of care by evaluating the performance of a healthcare service organization against a set of standards. Accredited healthcare service organizations are renowned for high-quality care and patient safety (1). The Joint Commission formed JCI to provide international clients with education and consulting services in 1994. JCI published its first international quality standards for hospitals in 2000. Our study focuses on JCI accreditation, which extends the Joint Commission’s mission and standards worldwide by helping healthcare services organizations outside the United States improve the quality of care and patient safety. Pursuing international accreditation is voluntary for any healthcare services organization in China. As of December 2022, forty-six Chinese healthcare services organizations accredited by JCI make China rank fifth on the list of countries with the highest number of JCI-accredited healthcare organizations and account for 5% of the total 946 JCI-accredited organizations worldwide. In China, forty-four private hospitals, one public hospital, and one homecare organization achieved and maintained JCI accreditation (2). Our study is the first qualitative study to explore Chinese hospital leaders’ perceptions toward JCI accreditation.

We used a few electronic databases, such as PubMed, Embase, and Cochrane Library, to review the literature about the perceptions of JCI accreditation. Keywords used in different combinations include “Joint Commission International,” “perception,” and “JCI accreditation.” The previous studies were conducted in certain countries, such as Saudi Arabia, Israel, Singapore, South Korea, Belgium, the United Arab Emirates, Panama, Lebanon, and Turkey. Research methods included the in-depth qualitative interview or a cross-sectional survey to explore the attitudes toward JCI accreditation from administrative staff, physicians, nurses, and other health professionals. JCI accreditation is a system approach that evaluates the capability of the entire healthcare organization to produce good results (3). Although JCI aims to extend the Joint Commission’s standards worldwide, there were complaints that JCI standards are less stringent than those of the Joint Commission in the United States (4). Previous studies revealed a need for more agreement about the value of JCI accreditation. Most nurses generally had positive attitudes, but most physicians perceived fewer benefits of participating in JCI processes than nurses (5). However, the characteristics of JCI were insufficient in attracting and retaining nurses (6). Compared with healthcare professionals, the administrative staff was more satisfied with implementing JCI standards (7). Evidence indicated perceived benefits of pursuing and implementing JCI accreditation, such as improving the quality of training and education (8), building up quality improvement and patient safety culture (9), reducing variation in medical care (10), valuing the organizational change (5), and enhance hospital branding (11). However, there was no consensus among hospital managers about whether or not JCI accreditation had an enduring impact on the improvement (9). Side effects of implementing JCI standards were perceived, such as the preparation of JCI distracted healthcare workers from daily clinical work (8), requiring substantial monetary resources (10), and being time-consuming (11).

Compared with the local hospital accreditation standards and procedures, hospital professionals are less familiar with JCI accreditation, and international standards. Pursuing JCI accreditation is a leadership decision, but few studies have explored senior leaders’ perceptions about JCI accreditation. The JCI accreditation standards have stricter requirements than the local accreditation standards. While JCI accreditation is unfamiliar to Chinese leaders and the standards more rigorous, Chinese leaders’ perceptions of the value of JCI accreditation would be consistent with study findings in other countries. Besides, our study may explore different motivations, challenges, and perceived benefits from Chinese hospital leaders’ perspectives.

## Materials and methods

### Design/methodology

Our study used a semi-structured interview approach to understand the perceptions of hospital leadership in JCI-accredited hospitals in China.

### Ethical approval

Prior to starting the study, ethical permission was obtained by the Institutional Review Board of the Johns Hopkins Bloomberg School of Public Health.

### Selecting participants

We applied the purposive sampling technique. Given a list of 40 JCI-accredited private hospitals in China, OBGYN hospitals accounted for 27.5% (11/40), the largest specialty hospital among accredited private specialty hospitals. The eligible participant would be the decision-maker in pursuing JCI accreditation and the leader in implementing JCI standards. Therefore, the leaders who hold any of the 3-key roles: Chief Operating Officer (COO), Chief Medical Officer (CMO), and Chief Quality Officer (CQO) were our targeted interviewees. After frequent contact, 15 leaders from five private OBGYN hospitals agreed to participate in in-depth interviews. The participants were given information that described the purpose of the study. They were asked to give oral informed consent to participate in the study before commencing the interviews. Table 1 lists the invited leaders and their positions. We labeled the interviewees by the hospitals and positions. For example, the COO at Hospital A is coded as COO1; the CMO at Hospital B is labeled as CMO2, and so on.

**Table 1:** Participant (*N* = 15) characteristics.

Hospital setting	Hospital A	Hospital B	Hospital C	Hospital D	Hospital E
Number of participants	3	3	3	3	3
Position					
Chief Operating Officer (COO)	1	1	1	1	1
Chief Medical Officer (CMO)	1	1	1	1	1
Chief Quality Officer (CQO)	1	1	1	1	1
Gender					
Male	1	2	1	1	1
Female	2	1	2	2	2
Highest level of education					
Bachelor	1	1	1	1	2
Master	1	1	0	1	0
Doctorate	1	1	2	1	1

### Data collection

An experienced qualitative researcher interviewed the participating executives at the hospital sites where they work, with two assistants during the process. The interviewer started by introducing themselves to the interviewee and asked permission to audio record the interview. The semi-structured questions (Table 2) were designed to elicit the participant’s perceived motivations, the challenges, and the benefits of JCI accreditation. The interviewer reordered the semi-structured questions depending on the participant’s flow of thought. The research assistants were responsible for taking notes about the conversation. The interview took 60 min on average.

**Table 2:** Semi-structured interview questions.

1	Is your organization JCI accredited?
2	Why is JCI accreditation important to your organization?
3	What are the motivations for your organization to pursue the JCI accreditation?
4	What are the challenges during the journey to JCI accreditation?
5	Can you describe the process for gaining buy-in to pursue JCI accreditation?
6	Has JCI accreditation improved your organization? 1) If so, please elaborate on examples 2) If not, what was expected?
7	Does your organization support JCI accreditation? Why or why not?
8	What are the benefits of the JCI accreditation program?
9	What are your attitudes toward the JCI accreditation?
10	What are your observations post-JCI’s survey in your organization?
11	What do you think about the difference between JCI and local standards?
12	What do you think are your colleague’s opinions on the JCI program?

### Data analysis

**Figure 1:**
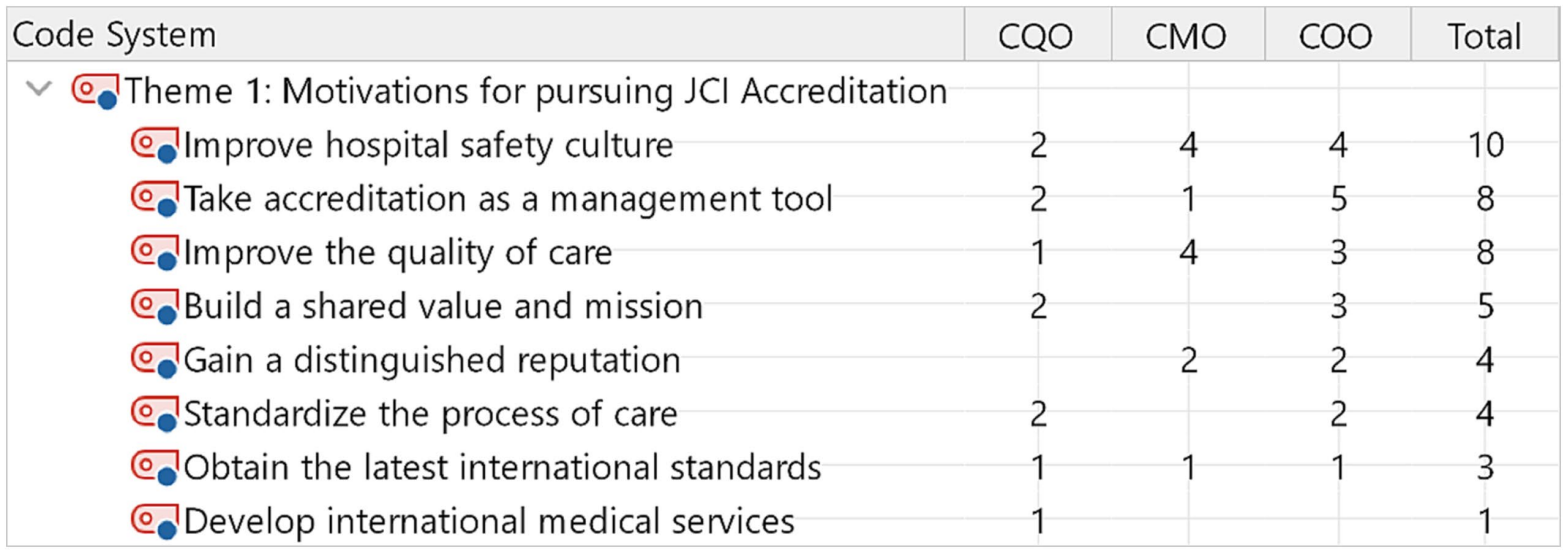
Code matrix browser within theme one.

**Figure 2:**
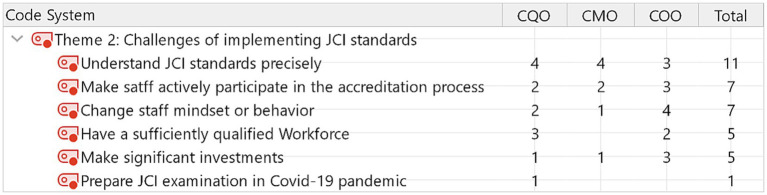
Perceived challenges of implenting JCI standards.

**Figure 3:**
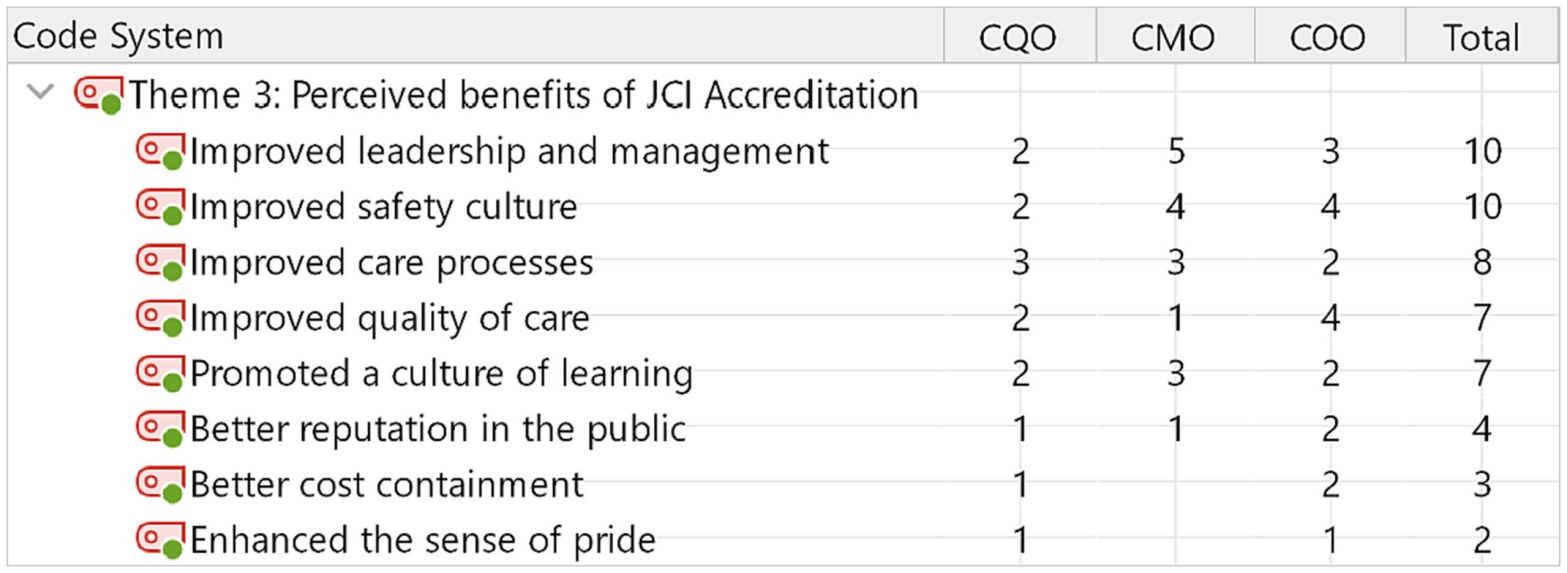
Perceived benefits of JCI accreditation.

The verbal data of the audio recording were transcribed into the written text of transcripts. The notes taken from the interview can be used as a second opinion on the accuracy of the recording. We followed Braun and Clarke’s six-phase process (12) and utilized MAXQDA 2022 for thematic analysis. Several stages (phases) are performed to do the analysis. First, we started the study by creating a new project on MAXQDA and importing the documents of interview transcripts into the new project. We read the entire data set a few times and identified semantic themes. Second, after understanding the data set, we used MAXQDA to code the data by tagging and naming text selections according to the features of the data. Then, we assigned codes to the data segments. Two-round coding was used to enhance the accuracy and coherence of coding. Two research assistants with training performed initial coding. An experienced qualitative researcher double-checked the codes. If inconsistency appears, a discussion will be used to reach an agreement. Third, we collated the relevant coded data extracts within identified themes. Within each theme, five to eight sub-themes were identified. Fourth, we re-read the entire data set to code any additional data within themes missed in earlier coding stages. Fifth, we finalized the themes and sub-themes by further refining those themes. Finally, with a set of fully worked-out themes, we completed the analysis, including suitable data extracts to demonstrate the prevalent themes. Visualizing the results is one of MAXQDA’s strengths, and Code Matrix Browser showed the results in Figures 1–3.

## Results

Our thematic analysis generated three main themes to describe Chinese hospital leadership’s perceptions of JCI accreditation. These are motivations for pursuing JCI accreditation, the challenges of implementing JCI standards, and the perceived benefits of JCI accreditation.

### Theme 1: motivations for pursuing JCI accreditation

The participants identified eight sub-themes related to perceived motivators to pursue accreditation. Ten out of fifteen participants expected JCI accreditation to improve the safety culture. Eight out of fifteen participants believed that JCI accreditation improved the quality of care and served as a management tool to improve performance. Figure 1 links sub-themes to types of professions. For example, chief medical officers expressed that JCI is motivated by improving hospital safety culture and improving the quality of care, in addition to improving hospital safety culture. In contrast, Chief operation officers recognized the motivation for JCI accreditation as a management tool.

Sample quotes from some participants in italics are as follows.

#### Use accreditation as a management tool

“I can use JCI standards to manage physicians’ behavior. Our physicians had different working experiences in other hospitals before they joined our hospital. Therefore, they had different behaviors or even mindsets in the process of medical care. I think JCI standards created a common language of care and patient safety among our physicians.” (CQO1)

“Our (Chinese) local hospital accreditation is a rigorous assessment, but it focuses on the medical outcomes. JCI accreditation evaluates the process of care but also assesses the hospital’s governance. We learned effective leadership in the JCI program.” (COO2)

### Theme 2: challenges of implementing JCI standards

Even though leadership decided to pursue the accreditation, implementing JCI standards into routine work inevitably encountered challenges. Among the six challenges of implementing JCI standards, 11 out of 15 participants (73%) expressed difficulty understanding JCI standards precisely. This appeared to be the common issue of transforming the English-based criteria to another language and medical care system. Figure 2 indicates the main concerns about implementing JCI standards. Making staff actively participate in the accreditation process and changing staff mindset or behavior is ranked as the second challenge to implementing JCI accreditation.

Sample quotes from some participants in italics are as follows.

#### Understand JCI standards precisely

“We knew little about JCI standards, which is a foreign accreditation. Thus, we engaged a consulting firm to help us prepare JCI examination. During the preparation of JCI accreditation, there were debates between the external consultants and our health care professionals about how and what to do to meet JCI requirements/standards.” (CMO1)

“We had to learn JCI standards on our own, but the Chinese version of JCI standards on our hands was difficult to understand. Our concern was the misunderstanding of JCI requirements” (CMO2)

“When some physicians complained that some of JCI requirements could conflict with local practices, we did not know who was able to make the judgment.” (COO1)

#### Change staff mindset or behavior

“Some physicians argued that local accreditation has the same level of requirements regarding improving the quality of care and patient safety, and therefore it was not necessary to pursue JCI accreditation” (COO3)

“Some staff got back to their old routines rather than strictly following JCI requirements in their daily works after the JCI survey (every three years) was passed.” (CMO3)

#### Unfavorable perceptions regarding JCI accreditation

One unfavorable perception is the relatively higher standard or criteria in physical and environmental safety policy, as compared with the minimum requirement required by China’s national or local requirements. Any infrastructure changes not only mean more money investment but also affect patients’ volume and revenue during the construction period. Some participants in our study recalled that at the time of the preparation for JCI accreditation, they were struggling to decide to renovate the hospital’s infrastructure and facilities to meet JCI requirements.

Another unfavorable perception was related to staff resistance to participating in JCI. One of the reasons behind the resistance could be the excessive documentation to meet JCI requirements. Both clinicians and other paramedical professionals complained that they have to spend more time on documentation, thereby taking some time from their patient care or patient-related work. In general, during the preparation, they need to work overtime to meet clinical work as well as the JCI documentation. Hospital executives encountered resistance from those staff who argued that some documentation was too excessive and unnecessary.

The third unfavorable perception happened during the JCI on-site examination. Few staff in hospitals can speak English fluently; however, JCI examiners are required to use English to interview staff. The hospital was responsible for having English translators during the on-site survey and interviews. The translators might not translate precisely both the questions or answers, which results in possible miscommunication or misunderstanding.

### Theme 3: perceived benefits due to JCI accreditation

Ten out of fifteen participants indicated that JCI accreditation improved hospital leadership management and safety culture (Figure 3). In general, CMOs and COOs perceived JCI benefits more than CQOs. Followed by leadership and safety culture, improved care processes, and quality of care, promoting a learning culture were recognized by the participating hospital leaders.

Sample quotes from participants in italics are as follows.

#### Improved leadership and management

“The chapter on GLD (Governance, Leadership, and Direction) in JCI Standards gave guidance on how to achieve effective leadership. Also, we can require teams to comply with a specific standard in the process of care by emphasizing that we worked in a JCI-accredited hospital.” (COO2)

“JCI standards helped optimize the existing processes. These effective processes can reduce waste and redundancies. I could not achieve the effective process alone. I have to more actively communicate with peers in different departments to find out what caused wastes, and then worked out solutions.” (CMO3)

#### Improved safety culture

“During the JCI program, we improved the mechanism for reporting an adverse event. We encourage our staff to identify potential risks and work out strategies and methods to prevent adverse events.” (CQO2)

## Discussion

The interrelated themes of JCI accreditation focused on the motivations for the change, challenges in the journey to the accreditation, and perceived benefits. The results indicated that Chinese hospital leaders were motivated by different goals when pursuing JCI accreditation. The prevalent motivations include using accreditation as a management tool to improve organizational performance, safety, and quality of care. However, the same leaders experienced difficulties in implementing the accreditation. The significant challenging tasks for the leaders are understanding the JCI standards precisely, making staff actively participate in the accreditation process, and changing their behaviors according to accreditation requirements.

There were a few relevant specific unfavored perceptions from leaders’ perspectives. Stricter JCI criteria in the hospital physical safety environment require the hospital to spend money to upgrade the hospital’s infrastructure and facilities and decrease the patient volume during the construction period. However, the investment would ultimately be helpful for patient safety, therefore differentiating the accredited hospital from the non-accredited hospital. Another study also found that resistance from medical staff was a significant issue in implementing JCI standards (5). This negative perception might be because team managers did not correctly understand the intention of individual JCI standards, and thereby, they could not know how to implement the JCI requirements effectively. Tarieh et al. (13) found that if hospital leaders ensure staff involvement with management decisions, staff motivation will increase. Then, staff resistance will be minimized, and staff productivity will increase (13). The increased unnecessary workloads lowered the staff’s enthusiasm for JCI-related tasks. For example, some staff complained that excessive documentation due to JCI requirements was unnecessary. Leaders should consider how the accreditation process may improve the documentation and decrease the staff’s workload. One of JCI accreditation’s characteristics could be its international, as its English standards are translated and applied in different countries and cultures worldwide. As few Chinese hospital staff have qualified English to understand foreign standards, translation is inevitable. Unqualified translation words could cause misunderstanding unless JCI reviewers can use the local language to conduct on-site surveys.

On the other hand, participants in our study perceived the benefits of JCI accreditation. The improved management leadership and safety culture were the two most benefits perceived by hospital leaders. De Meester et al. (14) indicated that JCI program improved the communication between the nurses and physicians, resulting in a lower number of unexpected deaths. Novarro-Escudero et al. (15) found that Under JCI guidelines, almost all stroke core measures continuously improved over three years. However, the observed improvements in accredited hospitals may not necessarily be attributed to the accreditation (16), and benefits to patient safety should not be resulted from hospital accreditation only (17). Getting international accreditation motivated some participants in our study, and they felt a distinguished reputation when their hospitals achieved JCI accreditation. This result is consistent with the finding that although healthcare professionals felt stressed about achieving the accreditation, they were proud after they went through the accreditation process (18). However, one study suggested that achieving accreditation cannot guarantee that the accredited hospital provides high-quality care continuously (18).

Our study’s implications are consistent with previous studies. First, patient safety and quality of care is the most prominent benefit of JCI accreditation as perceived by leaders. Therefore, from a macro level, in the context of upgrading a national healthcare system to achieve a good quality of care, the study findings may provide references to policymakers, as well as hospital management decision-makers, to consider JCI accreditation as a management tool. Good leadership and organizational culture facilitate JCI accreditation implementation (19). Top management is vital for successful implementation and effectiveness (20). Hospital leadership commitment and effective governance are essential to effectively implementing JCI accreditation (21). Financial support is needed to have qualified infrastructure, equipment, and staff education to comply with JCI requirements. Staff resistance was seen as a big challenge in implementing accreditation. One possible reason for resistance could be that there were no monetary or other incentives to encourage healthcare professionals or administrators to participate in the JCI program. Another study pointed out that the resources to achieve accreditation should be based on support from coworkers and managers (22). The findings in our study implied that leaders are responsible for leading staff across different departments to collaborate to achieve accreditation by allocating monetary and human resources in the accreditation program. Although the implementation of accreditation requires all staff participation in the hospital, its leaders should be accountable for driving the process (18). If the hospital leadership showed commitment to the process, the implementation of accreditation would be easier, especially when hospital leaders involved staff in the process (7). In addition to commitment in the process, another way to overcome the resistance issue is the education or dissemination of JCI’s impact on medical care quality, as well as timesaving for workers’ daily routine, which might reduce the resistance toward JCI accreditation. To address the challenge of language barriers, the hospital’s top executives might implement a periodic review of the JCI accreditation standards or procedures to enhance the comprehension of the standard in a selected group of members.

Our survey participants are limited to the hospital’s top management; their perception is not subjected to the opinion of the first-line managers or employees. In general, first-line healthcare workers are persons who carry the daily and prepare or execute the JCI policy and procedures. The future study is suggested to interview to solicit perceptions of physicians, nurses, and first-line managers. We will develop a research questionnaire based on the in-depth interview results and survey the same hospital workers to examine their perceptions on JCI accreditation.

## Limitations

As the study was limited to a few hospitals in China, the results could not be relevant to other settings. However, the study aimed to understand Chinese hospital leaders’ perceptions, thereby adding to the current literature. Another limitation is that the study only included private hospitals, and the selection bias made the findings in the study not generalizable to public hospitals. However, the public hospital only accounts for 2% of JCI-accredited hospitals in China. Public hospitals in China are less pursuing the JCI accreditation than the private hospital’s counterpart, as quality improvement strategy. Although hospital numbers are about equal in both sectors, the scale of public hospitals is larger than that of private hospitals. The complexity of management increase with the number of bed. The study only limited to private hospital leaders’ perception; if a similar study is conducted at public hospitals, a comparison could be derived for the larger healthcare system. The third limitation is that the number of participants in our study is small; they could not represent the leadership in other private hospitals. However, the participants provided rich information about JCI accreditation initiatives and implementation, consistent with the literature. However, the participants who accepted the interviews might favor the JCI accreditation policy over those who refused to participate in the study (23).

## Conclusion

The impact of JCI accreditation has been widely studied in different countries, but few qualitative studies have been conducted in Chinese hospitals. Our paper is the first study to explore the perceptions of leadership in JCI-accredited hospitals in China on motivation, challenges, and benefits of JCI accreditation. Despite its limitations, the study contributed to the current literature. Our study indicated that the leaders in Chinese private hospitals value JCI accreditation as a management tool based on patient-centeredness to better treat individual patients and their family members. The same organization leaders encountered one of the challenges in leading the JCI program: staff resistance. One of the reasons behind the opposition could be the issue of interpreting JCI standards. Not properly understanding JCI requirements would cause unnecessary workload and waste resources. It lowered staff enthusiasm to participate in the accreditation. Also, our study added to the understanding of leaders’ thoughts when they planned the accreditation and their experiences during the implementation of accreditation. These findings provide insight that dealing with those difficulties during the accreditation process may improve leadership and hospital culture.

## Data availability statement

The raw data supporting the conclusions of this article will be made available by the authors, without undue reservation.

## Ethics statement

Ethical permission was obtained from the Institutional Review Board (IRB) at the Johns Hopkins Bloomberg School of Public Health (reference number IRB00021496). The participants were given information that described the purpose of the study. They were asked to give oral informed consent to participate in the study before commencing the talk.

## Author contributions

HZ: Conceptualization, Formal analysis, Writing – original draft. ST-H: Data curation, Writing – review & editing. MB: Supervision, Writing – review & editing. LE: Writing – review & editing. HC-C: Conceptualization, Methodology, Writing – review & editing.
